# Acupuncture for lateral epicondylitis (tennis elbow): study protocol for a randomized, practitioner-assessor blinded, controlled pilot clinical trial

**DOI:** 10.1186/1745-6215-14-174

**Published:** 2013-06-14

**Authors:** Kyung-Min Shin, Joo-Hee Kim, Seunghoon Lee, Mi-Suk Shin, Tae-Hun Kim, Hyo-Ju Park, Min-Hee Lee, Kwon-Eui Hong, Seungdeok Lee, Sun-Mi Choi

**Affiliations:** 1 Department of Medical Research, Korea Institute of Oriental Medicine, Daejeon, South Korea; 2 Clinical research center, MokHuri Neck&Back Hospital, Seoul, South Korea; 3 Department of Acupuncture & Moxibustion Medicine, College of Korean Medicine, Daejeon University, Daejeon, South Korea; 4 Department of Acupuncture & Moxibustion Medicine, College of Korean Medicine, Dongguk University, Gyeongju, South Korea

**Keywords:** Acupuncture, Tennis elbow, Epicondylitis

## Abstract

**Background:**

Lateral epicondylitis is the most frequent cause of pain around the elbow joint. It causes pain in the region of the elbow joint and results in dysfunction of the elbow and deterioration of the quality of life. The purpose of this study is to compare the effects of ipsilateral acupuncture, contralateral acupuncture and sham acupuncture on lateral epicondylitis.

**Methods/design:**

Forty-five subjects with lateral epicondylitis will be randomized into three groups: the ipsilateral acupuncture group, contralateral acupuncture group and the sham acupuncture group. The inclusion criteria will be as follows: (1) age between 19 and 65 years with pain due to one-sided lateral epicondylitis that persisted for at least four weeks, (2) with tenderness on pressure limited to regions around the elbow joint, (3) complaining of pain during resistive extension of the middle finger or the wrist, (4) with average pain of NRS 4 or higher during the last one week at a screening visit and (5) voluntarily agree to this study and sign a written consent. Acupuncture treatment will be given 10 times in total for 4 weeks to all groups. Follow up observations will be conducted after the completion of the treatment, 8 weeks and 12 weeks after the random assignment. Ipsilateral acupuncture group and contralateral acupuncture group will receive acupuncture on LI4, TE5, LI10, LI11, LU5, LI12 and two Ashi points. The sham acupuncture group will receive treatment on acupuncture points not related to the lateral epicondylitis using a non-invasive method. The needles will be maintained for 20 minutes. The primary outcome will be differences in the visual analogue scale (VAS) for elbow pain between the groups. The secondary outcome will be differences in patient-rated tennis elbow evaluation (PRTEE), pain-free/maximum grip strength (Dynamometer), pressure pain threshold, clinically relevant improvement, patient global assessment, and the EQ-5D. The data will be analyzed with the paired *t*-test and ANCOVA (*P* <0.05).

**Discussion:**

The results of this study will allow evaluation of contralateral acupuncture from two aspects. First, if the contralateral acupuncture shows the effects similar to ipsilateral acupuncture, this will establish clinical basis for contralateral acupuncture. Second, if the effects of contralateral acupuncture are not comparable to the effects of ipsilateral acupuncture, but are shown to be similar to the effects of the sham acupuncture, we can establish the basis for using the same acupoints of the unaffected side as a control in acupuncture clinical studies.

**Trial registration:**

This trial has been registered with the ‘Clinical Research Information Service (CRIS)’, Republic of Korea: KCT0000628.

## Background

Lateral epicondylitis is a disease that can result in elbow joint pain and dysfunction, and decrease in the quality of life [1]. Lateral epicondylitis is the most common cause of pain around the elbow joint, and occurs because of overuse injury and repetitive stress [1,2]. Every year, about 1 to 3% of the general population is affected [3], and there is no difference in morbidity between the sexes, but the prevalence rate is higher in people over 40 years of age [4]. In addition, it is more frequent in the dominant arm [5], and if untreated the symptoms persist for 6 to 24 months [6-8].

Treatment for lateral epicondylitis includes non-steroidal anti-inflammatory drugs (NSAIDs), corticosteroid injection, botulinum toxin injection, extracorporeal shock wave therapy, low-level laser therapy, physiotherapy, acupuncture, and surgery [4]. The short-term effect of ultrasound therapy [9], low-level laser therapy [10], and acupuncture [11,12] has been accepted, but there is insufficient evidence regarding their long-term effects [13]. Further, the short-term effects of injection treatments have been accepted, but problems such as adverse events and high frequency of relapse, still exist [14-16].

Previous studies of lateral epicondylitis include a case study of fire needle therapy [17], and randomized controlled trials (RCTs) comparing the effects of acupuncture against sham acupuncture [18-20], physical therapy [21], and electro-acupuncture [22]. Sham acupuncture, which is used in clinical studies of acupuncture, includes invasive and noninvasive methods. The acupoint used as the control includes the actual acupoint used in the treatment, areas away from the actual acupoint (non-acupoint), or acupoints unrelated to the specific indication. Sham acupuncture used as controls in previous studies of lateral epicondylitis includes using areas away from the acupoint (non-acupoint) [18], minimal acupuncture at the same acupoint [20], and non-invasive acupuncture at an unrelated acupoint [19]. However, in clinical studies of acupuncture, one of the problems of using sham acupuncture is that practitioner blinding is not done.

Contralateral acupuncture is a traditional acupuncture technique, whereby acupuncture points on the right side are selected for diseases or disorder on the left and vice versa [23]. Several studies have shown effects of contralateral acupuncture on hemihidrosis [24], herpes zoster [24], dizziness [25], pain [24,26-29] and stroke [30]. Furthermore, there have been studies comparing the brain function [31], the change ratio of mean blood flux [32], and the blood volume [33] of the needle-inserted side and the side without insertion after insertion at acupoints, such as the LI4 and ST36, on normal subjects. However, there have not been any studies on lateral epicondylitis comparing ipsilateral acupuncture and contralateral acupuncture at the same acupoints.

Therefore, this study is a randomised, practitioner-blinded, parallel-group, sham-controlled pilot study of patients with lateral epicondylitis, to compare the efficacy of ipsilateral acupuncture, contralateral acupuncture, and sham acupuncture (non-invasive acupuncture at an unrelated acupoint). Especially, this study is designed to conduct a practitioner-blinded study by setting up separate groups of diagnosticians and practitioners in an attempt to examine the possibility of a practitioner-blinded clinical trial.

## Methods/design

### Aim of the study

The aim of this pilot study is to assess the efficacy of unaffected-side acupuncture by comparing the efficacy of ipsilateral acupuncture, contralateral acupuncture and placebo acupuncture in patients with lateral epicondylitis. And through this study, we will evaluate the possibility of a practitioner-blinded clinical trial.

### Study design

**Figure 1:**
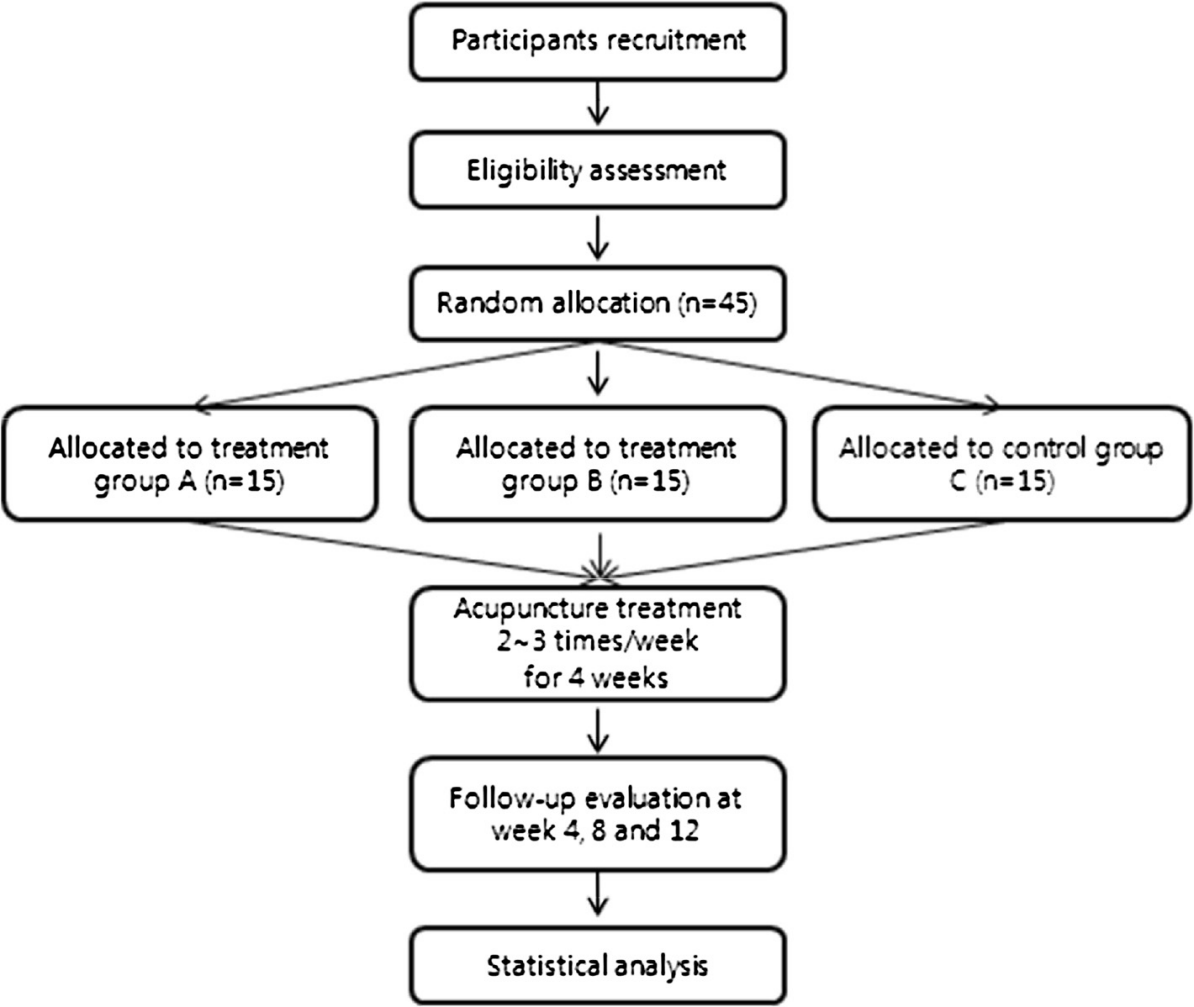
Flowchart of the study design.

This study is a randomised, practitioner-blinded (to affected side), parallel-group, placebo-controlled pilot study of patients with lateral epicondylitis. The subjects who voluntarily sign a consent form will undergo the examinations according to the clinical study design. When a subject is determined to be appropriate for participation based on the inclusion and exclusion criteria, the subject will be randomly assigned to one of three groups during the second visit (Table 1). Treatments will be performed two to three times a week. All three groups receive 10 acupuncture treatments over 4 weeks. A once-a-week treatment and a four-times-a-week treatment will be permitted to minimize the differences in the total number of treatments. Follow up observations will be conducted at the end of 4, 8 and at 12 weeks after randomized assignment. The affected- and unaffected-side acupoints were selected based on acupuncture literature and prior studies [18,20,21]. The acupoints for the control group were selected after referring to a previous study [19], with six acupoints added to maintain the same number of treated acupoints between the groups. To summarize, all three groups will receive acupunctures at eight acupoints (Figure 1).

**Table 1:** Patient inclusion and exclusion criteria

**Inclusion criteria**	**Exclusion criteria**
1. Individuals between the ages of 19 and 65 years with lateral epicondylitis on one arm and pain persisting for at least 4 weeks	1. Individuals whose radiological examinations show abnormalities such as calcification, arthritis, and inflammatory arthropathy of the elbow joint
2. Individuals with tenderness limited to the elbow joint and surrounding area	2. Individuals with a history of trauma, ligament damage, fracture, tumor, or surgery of the elbow joint
3. Individuals reporting pain under resisted extension of the middle finger and wrist	3. Individuals who have been diagnosed with or treated for cervical radiculopathy or herniation of intervertebral disc
4. Individuals with an average pain of 40 or more (0 to 100) on the visual analogue scale) in the week prior to the screening visit	4. Individuals who have received injections for lateral epicondylitis during the last 6 months
5. Individuals who volunteered to participate in the study and who signed a consent form	5. Individuals who have received treatments such as non-steroidal anti-inflammatory drugs, acupuncture, physiotherapy for lateral epicondylitis during the last 2 weeks
	6. Individuals judged by the person in charge of the clinical trial as unsuitable for participation, such as those with mental disorders, who are pregnant, or have other acute or chronic disorders

The practitioner and diagnostician have had more than 4 years of clinical experience since completing a 6-year-long college of Korean medicine and have received a Doctor of Korean Medicine.

### Randomization and allocation concealment

This study is designed as a pilot study and allowing for a 20% dropout rate, each group will include 15 participants [34,35]. Participants will be assigned with a 1:1:1 allocation ratio according to a computer-generated randomization list. Randomization will be stratified by dominant hand, as left- or right-handed. The allocation will be concealed in sequentially numbered, opaque, sealed envelopes and the random allocation envelops will be opened only after the participant has satisfied all selection criteria and completed baseline assessments. The participants and diagnostician will know the allocated group but the practitioners, outcome assessors and data analysts will not.

### Practitioner blinding

Practitioner blinding will be confirmed by separately involving diagnosticians and practitioners in the study. The practitioner will not participate in the screening and random assignment so will be unaware of the affected side. On screening and before each acupuncture treatment session, the diagnostician will assess the Ashi-points of the subjects. The diagnostician will mark and describe the subjects’ Ashi-points on a diagram of the arm, and then the practitioner will perform acupuncture after confirming only the side assigned for treatment. Appropriateness of practitioner blinding will be assessed after the end of the treatment.

### Ethics

This protocol adheres to the principles of the Declaration of Helsinki and has been approved by the institutional review boards of the Daejeon University Hospital, where the study will take place (djomc-100-1), and written consent will be obtained from each participant before any treatment is given. All patients will have the right to withdraw from the study at any time.

### Inclusion criteria

1. Individuals between the ages of 19 and 65 years with lateral epicondylitis on one arm and pain persisting for at least 4 weeks.

2. Individuals with tenderness limited to the elbow joint and surrounding area.

3. Individuals reporting pain under resisted extension of the middle finger and wrist.

4. Individuals with an average pain of 40 or more (0–100) on the visual analogue Scale (VAS) in the week prior to the screening visit.

5. Individuals who volunteered to participate in the study and who signed a consent form.

### Exclusion criteria

1. Individuals whose radiological examinations show abnormalities such as calcification, arthritis, and inflammatory arthropathy of the elbow joint.

2. Individuals with a history of trauma, ligament damage, fracture, tumor, or surgery of the elbow joint.

3. Individuals who have been diagnosed with or treated for cervical radiculopathy or herniation of intervertebral disc.

4. Individuals who have received injections for lateral epicondylitis during the last 6 months.

5. Individuals who have received treatments such as non-steroidal anti-inflammatory drugs (NSAIDs), acupuncture, physiotherapy for lateral epicondylitis during the last 2 weeks.

6. Individuals judged by the person in charge of the clinical trial as unsuitable for participation, such as those with mental disorders, who are pregnant, or have other acute or chronic disorders.

### Interventions

#### Ipsilateral acupuncture group

The subjects will receive 10 treatments for 4 weeks. Treatments will be performed two to three times a week, at a minimum of one per week and a maximum of four per week. The acupoints that will be used are as follows: LI4, TE5, LI10, LI11, LU5, LI12, and two Ashi-points. Sterilized disposable acupuncture needles of size 0.25 × 30 mm will be used. Deqi will be induced by manual stimulation, where the needles will be inserted for 20 minutes and then removed.

#### Contralateral acupuncture group

The subjects in the contralateral acupuncture group will receive the same frequency, duration, and total number of treatments as the ipsilateral acupuncture group. Needles will be inserted at the same acupoints of the unaffected side by using the same manual technique and retaining time.

#### Control group

For the control group, the acupoints used (BL13, Bl14, Bl15, and Bl16 of both the sides) are unrelated to the treatment of lateral epicondylitis, and non-invasive techniques will be used, namely, the Park sham placebo acupuncture device [36]. The subjects in the control group will receive the same frequency, duration, and total number of treatments and retaining time as the ipsilateral and contralateral acupuncture groups.

#### Prohibited concomitant treatments

During the treatment period, the subjects will be prohibited from receiving any concomitant treatments for the lateral epicondylitis. And the subjects will be allowed to decide whether to receive additional treatment during the assessment period. Any other related treatments will be recorded in detail.

### Outcomes

#### Primary outcome measure

The primary outcome measure in this study is the difference in visual analog scale (VAS) for elbow pain between the groups at 4 weeks after random assignment. The intensity of perceived pain of the elbow in the past week will be measured using a VAS consisting of a 100-mm horizontal line, where the left end indicates no pain, and the right end indicates maximum pain.

#### Secondary outcome measures

The secondary outcome measures are the difference in VAS for elbow pain between the groups at 8 and 12 weeks after random assignment, and the differences in patient-rated tennis elbow evaluation (PRTEE), pain-free/maximum grip strength, pressure pain threshold, clinically relevant improvement, patient global assessment and EuroQol between the groups at 4, 8, and 12 weeks after random assignment.

PRTEE, a tool specifically developed to assess lateral epicondylitis [37], will be used. PRTEE is composed of two parts: assessment of pain and function [38]. Pain-free/maximum grip strength will be measured using a dynamometer (Baseline® Digital Smedley Spring Dynamometer, Fabrication Enterprises, Inc., Irvington, New York, USA). The subjects will be asked to take a shoulder-width stance and allow their arms to hang loose. The pain-free grip strength will be measured, followed by the measurement of the maximum grip strength, and the affected side will be measured first and then the unaffected side. The measurement readings will be not revealed to the subjects until the completion of the test. The pain-free grip strength will be measured up to the point when the subject feels uneasy. The maximum grip strength will be measured at the maximum grip level. While administering a perpendicular pressure on the common extensor tendon area with a maximum pressure of 6 kg/cm^2^ by using a pressure algometer (PainTest™ FPX 25 Algometer, Wagner Instruments, Greenwich, CT, USA), the measurement will be taken at the moment when the sensation of pressure changes to pain. The mean value of three measurements taken at an interval of 20 seconds will be used [39]. Clinically relevant improvement will be defined when a 50% decrease in VAS is observed before and after the treatment. Quality of life will be measured using the Korean version of the EuroQol-5D (EQ-5D) tool [40]. Self-perceived improvements will be assessed by comparing sensations before and after the treatment using the patient global assessment (PGA) scale [41]. Subjects can select one of the following five responses to describe the improvement of symptoms when compared to that before the treatment: greatly improved; somewhat improved; no changes; somewhat exacerbated; and greatly exacerbated. A varied and translated version of the psychometric properties of the credibility/expectancy questionnaire [42] will be used to measure the credibility and expectancy of the subjects. From this questionnaire, the expectations of the subjects in relation to acupuncture therapy can be determined. Furthermore, differences in acupuncture efficacy depending on expectations can be assessed. In this study, we will be using the Southampton Needle Sensation Questionnaire [43] to assess the sensation that the subjects experience after the first acupuncture treatment. To determine whether practitioner blinding will be done appropriately, the practitioners will be asked to guess the group to which each of the subjects belongs.

#### Follow up

Follow up observations will be conducted at 8 and 12 weeks after the random assignment.

#### Statistical analysis

Analysis of covariance (ANCOVA) will be performed, with the primary analysis variables and relative changes in the values of secondary analysis variables, at the 4th, 8th and 12th week, compared to the baseline values, as dependent variables. The baseline value will be set as a covariate and the group as the fixed factor. The statistical significance threshold will be set at 0.05 (two-sided), with the 95% confidence interval. Missing data will be handled with the mixed model for repeated measures (MMRM) under the assumption that observations are missing at random (MAR). Safety analysis will be performed by analyzing the frequency of adverse events suspected as related to the treatment, and serious adverse events. The data for adverse events will be collected through the symptoms reported by the patients, and observations by a researcher at every visit. Among the variables for the primary outcome, sub analysis can be performed to determine whether the pain area of the subjects, faith in acupuncture treatment, and credibility of the efficacy of acupuncture treatment affect actual improvements in pain. Regression analysis can be performed to find the variables that have an influence on pain reduction. We will use the SAS® version 9.3 (SAS institute. Inc., Cary, NC, USA) for statistical analysis.

#### Adverse events

The subjects will be requested to voluntarily report information about adverse events, and the researcher will confirm the occurrence of adverse events through methods such as a medical interview. Details about adverse events, such as the date of occurrence, lost time, degree of adverse events, measures taken related to the treatment, causal relationship with the treatment, other treatments or medications that are suspected to cause the adverse event, and treatment of the adverse event, will be recorded in detail.

## Discussion

By using the results of our study, we plan to evaluate two aspects of contralateral acupuncture. First, if the contralateral acupuncture shows similar efficacy to the ipsilateral acupuncture, we can establish the clinical basis for contralateral acupuncture. Second, if contralateral acupuncture is not as efficacious as ipsilateral acupuncture and is similar to sham acupuncture, then we can establish the basis for using the same acupoints of the unaffected side as a control in acupuncture clinical studies. In other words, in diseases where either one of the left or right sides is affected, such as diseases of the shoulder joint, elbow joint, knee joint, or ankle joint, the contralateral acupuncture could serve as areas where sham acupuncture can be performed and can allow double-blinded studies.

## Trial status

This trial is currently recruiting participants.

## Abbreviations

ANCOVA: Analysis of covariance; EQ-5D: EuroQol-5D; MAR: Missing at random; MMRM: Mixed model for repeated measures; NSAID: Non-steroidal anti-inflammatory drug; PGA: Patient global assessment; PRTEE: Patient-rated tennis elbow evaluation; RCT: Randomized controlled trial; VAS: Visual analog scale

## Competing interests

The authors declare that they have no competing interests.

## Authors’ contributions

KMS, SDL, and KEH participated in the design of the study, coordinate the study and drafted the manuscript. JHK, SHL, MSS, THK, HJP, MHL, and SMC provided technical advice and wrote the relevant sections of the manuscript. All authors participated in, read, and approved the final manuscript.
